# Conservation and implications of eukaryote transcriptional regulatory regions across multiple species

**DOI:** 10.1186/1471-2164-9-623

**Published:** 2008-12-20

**Authors:** Lin Wan, Dayong Li, Donglei Zhang, Xue Liu, Wenjiang J Fu, Lihuang Zhu, Minghua Deng, Fengzhu Sun, Minping Qian

**Affiliations:** 1 School of Mathematical Sciences, Peking University, Beijing 100871, PR China; 2 Center for Theoretical Biology, Peking University, Beijing 100871, PR China; 3 State Key Laboratory of Plant Genomics and National Center for Plant Gene Research, Institute of Genetics and Developmental Biology, Chinese Academy of Sciences, Beijing 100101, PR China; 4 Department of Epidemiology, Michigan State University, East Lansing, Michigan 48824, USA; 5 MOE Key Laboratory of Bioinformatics and Bioinformatics Division, TNLIST/Department of Automation, Tsinghua University, Beijing 100871, PR China; 6 Molecular and Computational Biology Program, University of Southern California, Los Angeles, California 90089, USA

## Abstract

**Background:**

Increasing evidence shows that whole genomes of eukaryotes are almost entirely transcribed into both protein coding genes and an enormous number of non-protein-coding RNAs (ncRNAs). Therefore, revealing the underlying regulatory mechanisms of transcripts becomes imperative. However, for a complete understanding of transcriptional regulatory mechanisms, we need to identify the regions in which they are found. We will call these transcriptional regulation regions, or TRRs, which can be considered functional regions containing a cluster of regulatory elements that cooperatively recruit transcriptional factors for binding and then regulating the expression of transcripts.

**Results:**

We constructed a hierarchical stochastic language (HSL) model for the identification of core TRRs in yeast based on regulatory cooperation among TRR elements. The HSL model trained based on yeast achieved comparable accuracy in predicting TRRs in other species, e.g., fruit fly, human, and rice, thus demonstrating the conservation of TRRs across species. The HSL model was also used to identify the TRRs of genes, such as p53 or *OsALYL1*, as well as microRNAs. In addition, the ENCODE regions were examined by HSL, and TRRs were found to pervasively locate in the genomes.

**Conclusion:**

Our findings indicate that 1) the HSL model can be used to accurately predict core TRRs of transcripts across species and 2) identified core TRRs by HSL are proper candidates for the further scrutiny of specific regulatory elements and mechanisms. Meanwhile, the regulatory activity taking place in the abundant numbers of ncRNAs might account for the ubiquitous presence of TRRs across the genome. In addition, we also found that the TRRs of protein coding genes and ncRNAs are similar in structure, with the latter being more conserved than the former.

## Background

The identification of transcriptional regulatory elements that control the expression of each transcript is a fundamental and challenging problem in biology. Although tremendous progress has been made, both experimentally and computationally, the regulatory elements of genes are still not well understood, and only a handful of them have so far been experimentally verified [1]. The computational identification of regulatory elements is difficult for several reasons. First, genomic regulatory elements are usually short and degenerate [2]. Second, they are usually distributed across large genomic regions from the distant 5' upstream regions to the 3' downstream regions [3]. Meanwhile, ENCODE, a pilot project to identify all functional elements in the human genome sequence, has revealed that at least 93% of the human genome is transcribed in different cells and that regulatory sequences are symmetrically distributed around transcription starting sites (TSS) [4]. Thus, when searching these consensus sequences of transcriptional factor binding sites (TFBSs), typically 6 to 10 bases, we may obtain a large number of matches, but the majority of them have no relevant biological functions.

Many mechanisms controlling gene expression, including alternative TSSs and non-coding and antisense transcriptional controls, have been discovered [4,5]. However, most genes are controlled cooperatively by several transcription factors (TFs) binding to various regulatory elements [6-8]. These cooperating regulatory elements are often located in close spatial proximity to each other. Such co-appearance of regulatory elements can potentially help in the identification of transcriptional regulatory elements and regions. In this paper, we aim to identify and characterize such functional regulatory DNA regions. We term these regions transcriptional regulatory regions (TRR) and further define them as containing a cluster of cooperating regulatory elements which can recruit transcriptional factors for binding and then regulating the expression of transcripts. Once a TRR is identified, we show that further experiments can be conducted to elucidate its regulatory roles. Different from the widely recognized promoters (e.g., core promoters and proximal promoters), which are defined as DNA regions surrounding a specific location (e.g., TSS) [9], TRRs include not only functional promoter regions as a subset which participate in driving initiation of transcription, but also functional DNA regions located in introns, exons, or other intergenic regions, far away from TSSs, but still exerting their implicit regulatory roles.

Since our present understanding of TRRs is limited and primitive, we need an enhanced biological context to improve our understanding. It has been known for years that organisms as diverse as human, rat, *Drosophila*, and yeast use the same set of conserved proteins to initiate mRNA synthesis and that these proteins are collectively known as general transcription factors [10]. Therefore, we propose the concept of core TRR, which refers to a DNA region containing a cluster of conserved regulatory elements commonly occurring in the majority of TRRs. Using this principle for the present study, we first focused on predicting the core TRRs and then illustrated how these core parts are common for different transcripts and conserved across species.

By incorporating the idea of TF regulatory cooperation, substantial work has been done in the identification of regulatory regions *in silico*. For instance, Wasserman and Fickett first proposed the concept of *cis*-regulatory module (CRM) [6]. CRM refers to a DNA segment, typically a few hundred base pairs in length, containing multiple binding sites which recruit several cooperating transcription factors to a particular genomic location at a particular condition. Methods of identifying CRMs have been studied intensively and have been applied to many different settings [11-16]. Most studies were based on sets of potentially co-regulated genes selected by using various approaches, including, for example, gene expression arrays and ChIP-chip data. These approaches mostly aim to identify CRMs for specific biological processes and/or functions [17].

In contrast, this study aims to predict TRRs, as defined above, directly from the genomic sequences without additional (a priori) information, such as the sets of co-regulated genes used as inputs of algorithms for CRM. The main difference between core TRRs and CRMs is that CRMs are characterized by a module consisting of function/gene-specific elements, while the core TRR is a cluster consisting of the common and conserved parts of TRRs. In fact, the identification and characterization of core TRRs are made possible by their conservation. We have found, moreover, that these conserved regulatory elements are often shared by many kinds of TRRs for various biological processes and functions. This study compares the possible regulatory regions of various genes with random sequences and/or coding sequences in order to identify core TRRs. For simple model organisms, such as *S. cerevisiae*, intergenic regions can be taken as potential TRRs since they are relatively short (with median length of 400 bp), and most of them have regulatory roles [1]. Even though intergenic regions in most eukaryotic organisms are generally very large and not well understood, we show that the core TRRs are conserved among genes, not only in one organism, but also across species.

To accomplish this, we developed the hierarchical stochastic language (HSL) model which was previously used to identify vertebrate promoters [18]. The hierarchical structure model was also employed to identify CRMs [12]. However, in this study, we extended the HSL model to identify core TRRs based on a set of putative TRRs in yeast (positive set) and a negative set, either of coding DNA sequences or of randomly generated sequences. The HSL model first identifies a set of k-tuples (k = 6 in this study) that are significantly over-represented in the positive set versus the negative set. Pairs of k-tuples selected in the first step that are over-represented in the positive set compared to the negative set are then further identified. A classifier is then built to identify core TRRs in any given genomic DNA region. The detailed description of the HSL model is provided in the **Methods **section.

We first applied the HSL model to *S. cerevisiae *in order to build a dictionary of vocabularies (tuple-pairs). It is remarkable to observe that the vocabularies defined by *S. cerevisiae *were conserved across species such that the model trained based solely on *S. cerevisiae *can be applied to other species with similar accuracy. We then applied the HSL model to identify TRRs of several genes, including the human p53 gene, the rice gene *OsALYL1*, and others. In addition, by applying our HSL algorithm to 50 experimentally verified promoters of microRNAs in Arabidopsis [19], our HSL model achieved accuracies similar to those in protein coding genes for the identification of TRR regions in upstream sequences, indicating that the core part for transcription of microRNAs is similar to that of protein coding genes. In addition, after applying HSL to ncRNAs in ENCODE human genome regions, we found that TRRs are located pervasively in the genomes and that most TRRs might be responsible for the regulation of ncRNAs. The TRRs of ncRNAs are more conserved than those of protein coding genes. Therefore, by using TRR analysis, this work provides important biological insights into gene regulation.

## Results

We developed an HSL model for detecting core TRRs. We first showed that the HSL model based on yeast can be effectively used to predict TRRs of other organisms, including fruit fly, human and rice. We next applied the HSL model to several other cases, including (1) core TRRs of the p53 gene and genes potentially regulated by p53; (2) prediction of core TRRs for a new rice gene, *OsALYL1*, with experimental validations; (3) core TRRs of microRNAs of *Arabidopsis thaliana*; (4) core TRRs of the sense/antisense gene pair P5CDH and SRO5 of *Arabidopsis thaliana*; and (5) core TRRs in non-coding human DNA sequences.

### Building the HSL model using *S. cerevisiae *sequences

The HSL model was applied to the identification of core TRRs of *S. cerevisiae*. The model was trained using 2961 putative core TRR sample sequences (positive samples) extracted from the regions (-500, +100) of these genes on chromosomes I-XII. Their initial ATG codon was used as the origin for two reasons. The open reading frame (ORF) is well annotated in the Saccharomyces Genome Database (SGD), and the initial ATG codon of yeast genes has also been used to evaluate signals such as histone modification and nucleosome positions [20,21]. In contrast, *S. cerevisiae *coding DNA sequences (CDSs) and random DNA sequences (RDSs) were used as the negative samples. A primary dictionary of 534 k-tuples (k = 6), over-represented in the positive samples and under-represented in both CDSs and RDSs, was constructed with a likelihood ratio score for each k-tuple. Similarly, a second level dictionary of 3070 pairs of k-tuples from the primary dictionary was further constructed. Since we used both CDSs and RDSs as negative samples, we hypothesized that the dictionary should be able to distinguish TRR sample sequences from both CDSs and RDSs. For a given DNA sequence, a score based on the tuple-pair (second level) dictionary was given for each window of L base pairs along the sequence to indicate the likelihood of core TRR, where L can be chosen based on the required resolution, and we used L = 300 in this study. A threshold can be given to distinguish the core TRRs from non-core TRRs. Table 1 shows the top 100 (3.26%) high-scored tuple-pairs from the 3070 pairs. From Table 1, we can see that most of the top-scored tuple-pairs contain the TATA box-like motifs. We can also see some poly-T "TTTTTT" and poly-A "AAAAAA" words in the selected pairs. It is interesting to observe that poly-T and poly-A words are also over-represented in human promoter within the top 3 ranks [22]. Other motifs, such as CAAT box-like motifs, can also be found in the tuple-pair dictionary (out of the top 100 ranks; not shown in Table 1).

**Table 1:** Top 100 high-scored tuple-pairs from tuple-pair dictionary.

1–21	21–41	41–60	61–80	81–100
TATATA-TTTTTT	TATATA-GTTTTT	ATATAA-CTTTTT	CATATA-CTTTTT	CTTTTC-TTTTTT

TATATA-TTTTTC	TATATA-TTTCCT	CATATA-TTTTTT	TATATA-AGTTTT	TCTTTT-CTTTTT

TATATA-ATATAT	TATATA-TATTTT	GTATAT-CTTTTT	CCCTTT-TTTTTT	ATATAA-TTTTCT

TATATA-TTTCTT	TATATA-ATTTTT	TATATA-TTGTTT	TTTTTC-CTTTTT	TATATA-AAAAAG

TATATA-TTTTTA	TATATA-AAAGTA	ATATAC-TTTTTT	ATATAC-CTTTTT	TATATA-TTTGTT

TATATA-CTTTTT	TATATA-ATATAC	ATATAA-ATATAT	TATATA-ATATTT	CATATA-TCTTTT

CATATA-TATATA	CTTTTT-TTTTTT	TATATA-TGTTTT	TTTCTT-TTTTTT	TTCTTT-TTTTTT

ATATAA-TATATA	CATATA-ATATAT	CATATA-TTTTTC	TATATA-ACTTTT	ATATAA-TCTTTT

TATATA-TTTTCT	ATATAA-TTTTTT	ATATAA-TTTTTC	ATATAT-GTATAT	ACATAT-TTTTTT

TATATA-TCTTTT	AATAAA-TATATA	TCTTTT-TTTTTT	AAAATA-TATATA	TATATA-TTTTTG

TATATA-CTTTTC	TTTTTC-TTTTTT	TATATA-CATTTT	ATAAAA-TATATA	CATATA-TTTTCT

TATATA-TTCTTT	TATATA-TCCTTT	TATATA-TATTTC	TTTTTC-TTTTCT	CCTTTT-TTTTTT

TATATA-CCTTTT	TATATA-TTATTT	AAATAG-TTTTTT	TATATA-TAAAAG	CTTTCT-CTTTTT

TATATA-GTATAT	TTTTCT-TTTTTT	TTTCCT-TTTTTT	TTTTTC-ATATAT	TTTTCT-TTTCTT

TATATA-TTTTCC	TATATA-TTTTAT	ATATAA-GTATAT	CTTTCT-TTTTTT	TTTTTC-CCCTTT

TATATA-TTCCTT	TTTTCA-TATATA	TATATA-TTTATT	CCTTTT-CTTTTT	ATATAT-TTTTCT

AAATAA-TATATA	TATATA-TATTCT	TAAAAA-TATATA	TCTCTT-TTTTTT	CTTTTC-CTTTTT

TATATA-ACATAT	TATATA-TTCTTC	ATATAC-ATATAT	ATATAT-CTTTTT	TATATA-ATTGTT

TATATA-TATATT	ATATAT-TTTTTT	TATATA-ATTTTC	ATATAA-TTTCTT	GTATAT-TTTTTT

TATATA-TTTTGT	AAAAAA-TATATA	TATATA-ATTCTT	TTTTTC-TCTCTT	TTTTTC-AAATAG

### Genomic analysis of core TRRs in *S. cerevisiae*

**Figure 1:**
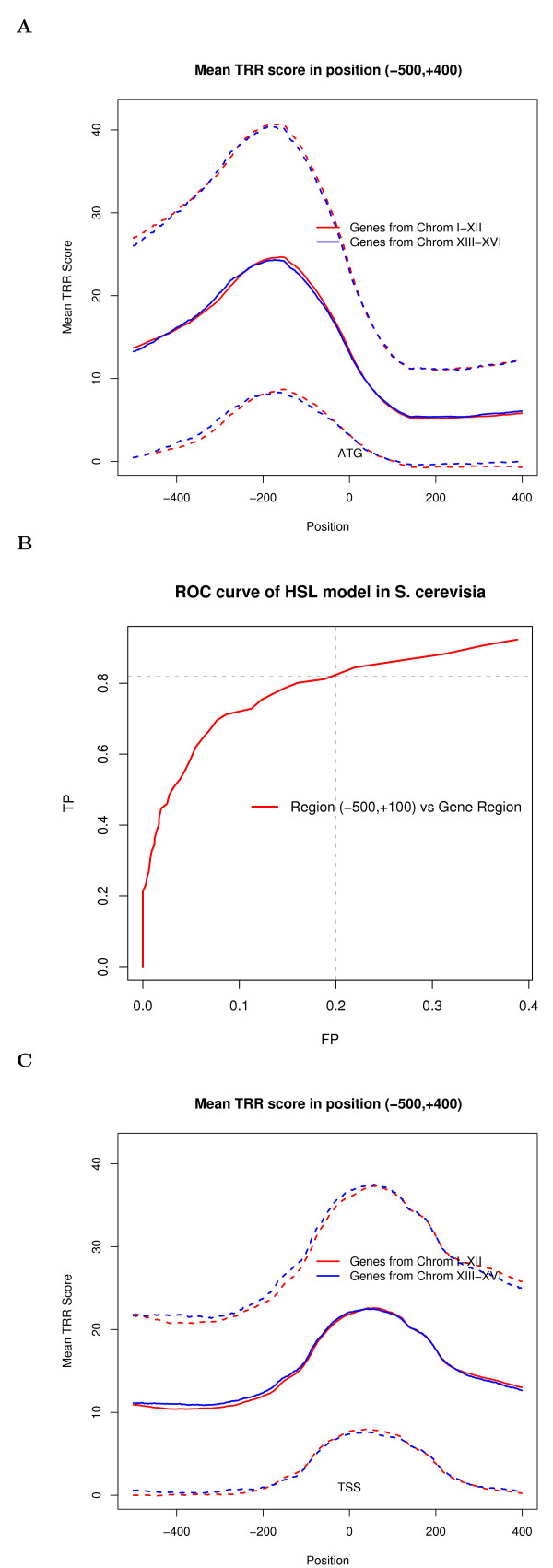
**Core TRRs analysis for yeast**. (A) The average score of 1317 genes on chromosomes XIII-XVI with annotation "Verified" and with ORF regions of more than 400 bp from *S. cerevisiae *for each position in (-500, +400) (blue curve). The red curve is the same as the blue curve, but using genes on chromosomes I-XII with annotation "Verified" and with ORF regions of more than 400 bp from *S. cerevisiae*. The "0" in the x-axis indicates the initial ATG codon. The dashed curves are generated by plotting the mean TRR score of each position ± 1 standard deviations of each position. (B) ROC curves for HSL model in yeast. The x-axis represents the false positive (FP) rate; the y-axis represents the true positive (TP) rate. (C) The same as (A) except we aligned genes by their TSSs. The average score of genes on chromosomes XIII-XVI with annotation of TSSs from [23] (blue curve). The red curve is the same as the blue curve, but using genes on chromosomes I-XII. The "0" in the x-axis indicates the TSS. The dashed curves are generated by plotting the mean TRR score of each position ± 1 standard deviations of each position.

We studied the distribution of TRR scores of verified genes in yeast. A total of 1317 genes on chromosomes XIII-XVI with annotation "Verified" and ORF regions of more than 400 bp were selected from *S. cerevisiae*. We aligned these 1317 sequences using their initial ATG codon as origin and calculated the average TRR scores for each aligned position. The results are shown in Figure 1A. The average scores peak in the region around -180 and decrease sharply in the downstream of ATG codon (blue curve in Figure 1A). Similar results were achieved for genes from training samples on chromosomes I-XI (red curve in Figure 1A). It is well known that promoters tend to be located in nucleosome-free regions, and a recent study [20] showed that the region around -180 has the lowest average nucleosome-occupancy signal. This result is consistent with our finding that the region around -180 is enriched for TRRs. In addition, the standard deviation of TRR scores generally increases with the mean TRR score (Figure 1A), possibly resulting in a different TRR score curve for individual genes.

Furthermore, we tested if the HSL model could distinguish TRR-containing regions from those not containing TRRs. We chose the region (-500, +100) of the 1317 genes as positive samples. We also generated the same number of negative samples with the same length as the positive samples by randomly sampling from gene regions on chromosomes XIII-XVI. For each sequence, if the maximum of the TRR scores along either sense or antisense strand is higher than a given threshold, the sequence is predicated to contain core TRRs. The true positive (TP) rate is taken as the percentage of predicated TRRs in positive samples, while the false positive (FP) rate is the percentage of predicated TRRs in the negative sample set. By setting different thresholds, a receiver operating characteristic (ROC) curve for our prediction model in yeast can be obtained and is shown in Figure 1B. When FP is set at 20%, the TP rate for yeast is 82%. Further studies of the relationship between TRR scores and gene transcriptional rates suggested a reasonable threshold score of TRR between 15 and 20 (see Additional file 1). Using a threshold score of 20 for core TRRs, our algorithm predicted 1163 (88.3%) out of the 1317 genes with core TRRs in the region (-500, +100) with FP rate of 33.9%. Among them, 1022 genes and 141 genes were detected on sense and antisense strands, respectively.

It should be noted that Lee et al. generated 5,015 TSS annotations for yeast genes with high-confidence [23]. We aligned genes by their TSSs from [23] and plotted the mean TRR score for each position (see Figure 1C). As Figure 1C shows, we found almost the same curves as seen in Figure 1A, except that the peak of Figure 1C shifts to 0 of the x-axis (the location of TSS), while for Figure 1A, the peak locates around 180 bp upstream of ATG.

### Core TRRs are conserved across eukaryote species

We next directly applied the HSL model trained by the sample sequences of *S. cerevisiae *to predict core TRRs of *Drosophila melanogaster *(fruit fly), *Homo sapiens *(human) and *Oryza sativa *(rice), and the model achieved similar accuracies.

We selected 1921 *Drosophila melanogaster *genes and 1844 *Homo sapiens *genes with experimentally verified TSSs from the Eukaryotic Promoter Database (EPD) [24], since these two are the only species for which over 1000 genes with experimentally verified TSSs in the EPD have been accumulated to date. In addition, the EPD database also contains TSS annotations for 13,044 *Oryza sativa *genes, albeit with less accuracy in the "Preliminary EPD entries" category. These 13,044 *Oryza sativa *genes were also included for the evaluation of the HSL model.

For each species, one set of positive sequence samples was formed with the EPD default (-500, +100) regions of the EPD genes (note that we used the TSS as their origin, not the initial ATG codon, which was used for yeast), and two sets of negative samples were constructed for separate tests: (1) randomly chosen coding DNA sequences of the same lengths as sequences in the positive sample and (2) randomly permuted sequences of each sample in the positive set. In this paper, unless otherwise specified, the sequences of negative samples are of the same length and the same number as the sequences of the corresponding positive samples.

Meanwhile, in order to evaluate the FP rates of HSL on intergenic regions, we generated a different kind of negative sample known as random intergenic sequences by Regulatory Sequence Analysis Tools (RSAT), a widely used tool which can generate sequences based on nucleotide composition of intergenic regions of each species [25,26]. Such randomly generated sequences were also used as negative control in promoter identification [27]. We did not use real intergenic regions as negative samples since such regions are not well studied and contain a large number of unknown TRRs [27,28]. However, for *Oryza sativa*, we did not have such negative samples because RSAT did not contain a trained rice-specific model.

**Figure 2:**
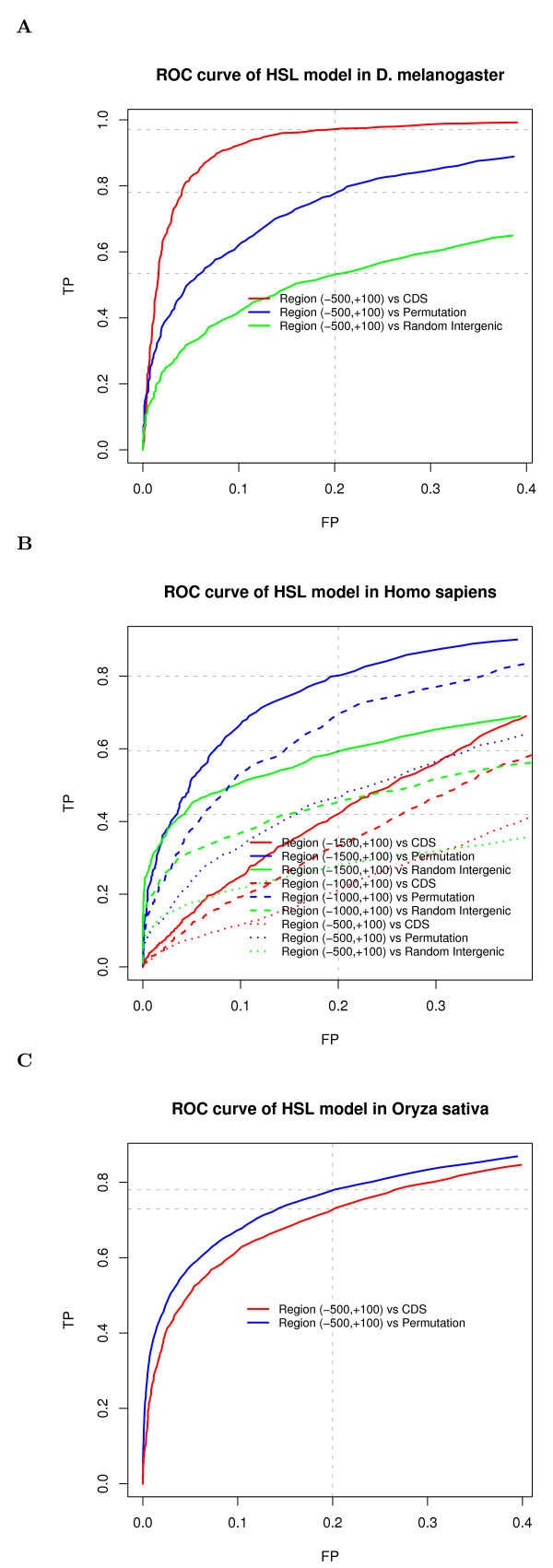
**ROC curves for HSL model in 3 testing species**: (A) *Drosophila melanogaster*; (B) *Homo sapiens*; (C) *Oryza sativa*. The x-axis represents the FP rate; the y-axis represents the TP rate.

Figure 2 shows the ROC curves of the HSL model in *Drosophila melanogaster*, *Homo sapiens *and *Oryza sativa*. In the case of the negative samples as protein coding DNA sequences, by fixing the FP rate at 20%, TP rates of *Drosophila melanogaster *and *Oryza sativa *are 97% and 73%, respectively; while, in the case of the negative samples as permuted TRR sequences, TP rates of *Drosophila melanogaster *and *Oryza sativa *are both around 78%. In the case of negative set as random intergenic sequences, the TP rate for *Drosophila melanogaster *is 54% when the FP rate is 20%.

However, TP rates of *Homo sapiens *in all 3 cases are below 50% with fixed FP at 20% (Figure 2B). The low human TP rate on the region (-500,+100) may have resulted from the complexity of human regulatory sequences and from the fact that many core promoters may locate farther away from the (-500, +100) regions of their corresponding genes [29]. Thus, we further extended the sequences of human positive samples to the upstream region; the corresponding sequences of negative samples were also generated with lengths equal to those in the positive sample set. From Figure 2B, we can see that, by extending the interrogating regions of samples, TP rates for human are markedly increased. When the region is extended to around (-1500,+100) and the FP rate is fixed at 20%, the TP rates for human reached 80% for the permuted negative samples, 60% for the randomly generated intergenic negative sample, but only 44% for the negative samples generated based on protein coding DNA sequence. The low TP rate in the case of protein coding DNA sequence as negative sample may be explained as follows. Multi-promoters and alternative splicing happen more frequently in human, and some TRRs may also locate in the coding areas. It has been shown that transcription not only starts in 5' UTR and 3' UTR, but it can also start in exons [30]. Thus, negative samples of protein coding DNA sequence may also contain TRRs, which could then result in overestimation of the FP. In addition, our studies were also restricted to the EPD regions (-100,+100), which are usually conserved and regarded as the sites of core promoters [9]. The results for human with biological implications can be found in the Additional file 1.

Nevertheless, these observations demonstrate that the basic elements of core TRRs are conserved across species and that the HSL model based on yeast can be used to predict core TRRs across different species. The conclusion is further supported by the successful application of the HSL model (trained based on *S. cerevisiae*) to identify TRRs of several genes in *Homo sapiens*, *Arabidopsis thaliana*, and *Oryza sativa L*.

### Core TRRs of the p53 gene

p53 is a vital transcription factor which regulates the expression of genes involved in a variety of cellular functions, such as apoptosis, cell cycle arrest, and DNA repair [31]. At least 80 proteins have been identified to bind the p53 gene, many of which can also influence its expression [32]. It has also been shown that the p53 gene has two promoters and can encode at least nine different p53 protein isoforms [33]. Transcription of the p53 gene can be initiated from two distinct sites upstream of exon 1 and from an internal promoter located in intron 4; intron 9 of the p53 gene can be alternatively spliced to produce three isoforms, including p53, p53-β and p53-γ [33,34].

**Figure 3:**
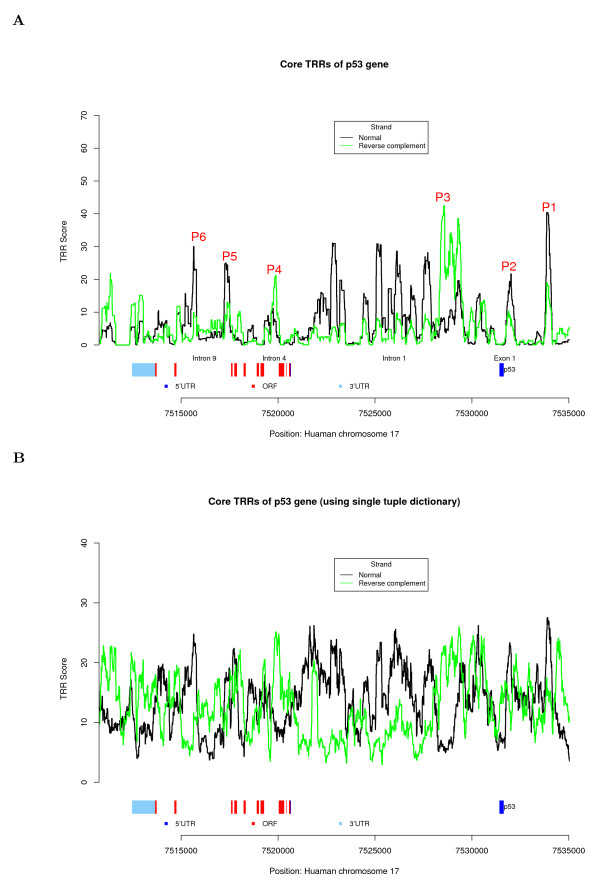
**Core TRRs analysis of the p53 gene in human**. (A) HSL model result of the p53 gene using tuple-pair dictionary. The x-axis represents the genomic positions of the p53 gene, and the y-axis represents the core TRR scores from the HSL model using tuple-pair dictionary from *S. cerevisiae*. "P1" to "P6" indicate the 6 TRR regions supported in the literature [33,34,36]. (B) HSL model result of the p53 gene using single tuple dictionary (534 k-tuples from *S. cerevisiae*).

We applied the HSL model, trained from yeast, to predict the core TRRs of the p53 gene and obtained the predicted scores shown in Figure 3A. [Here we used a stringent threshold, 20, which can achieve TP = 52.3%; FP = 26.7% for negative sample of protein coding DNA sequence, 5.21% for permuted sequences, and 12% for randomly generated intergenic sequences (Figure 2B)]. Comparing our TRR result (Figure 3A) with the experimental results of the p53 gene mentioned above, we can observe the following: (1) Two TRR signals ("P1" and "P2") are located within the 3000 bp upstream region of exon 1 of the p53 gene, and they correspond to the two distinct transcriptional initiation sites. (2) "P4" is the TRR signal located in intron 4 of the p53 gene, and it corresponds to the alternative promoter of p53 located in intron 4. (3) In intron 9 of p53, we identify the two TRR signals "P5" and "P6", and their regulatory functions need to be further studied.

The first intron of the p53 gene spans nearly 10 k bp with multiple predicated TRRs, a finding which agrees with previous discoveries, indicating that the first introns of genes in mammals tend to be longer than other introns and play an important role in regulating gene expression (see [35] for a recent summary). However, few concrete results have been reported on the functions of this region. In our predictions, "P3" is the strongest core TRR signal and is consistent with the recent available genomic landscape of histone modifications in human T cells [36]. Histone H3 K9/K14 diacetylation (H3K9acK14ac) and H3 K4 trimethylation (H3K4me3) have been reported to be co-localized with promoters and are associated with active genes required for T cell function and development [36]. The H3K9acK14ac and H3K4me3 signals of the p53 gene in T cells are located in intron 1 and co-localized with the predicted "P3". Other TRR signals in the first intron of p53 were further supported by chromatin modifications [37] and other information (see **Discussion **and Additional file 1). In summary, the signals in the first intron indicate some important regulatory events in this region.

### Core TRRs of genes regulated by p53

As a TF, p53 can target many other genes [38]. The genes controlled by p53 are expected to contain core TRRs near p53 binding loci to help p53 exert its functions. To test this hypothesis, we selected all 542 potential p53 binding loci (with median length of 1122 bp) in the human HCT116 cell line detected by ChIP-PET assay [39]. Out of these 542 loci, 392 (72.3%) had a significant core TRR signal (with a score of at least 20). Meanwhile, we also used 3 negative sample sets as negative controls: (1) equal number of protein coding DNA sequences of the same length as the identified p53 binding loci; (2) permuted sequences of p53 binding loci and (3) randomly generated human intergenic sequences by RSAT. We found that FP rates were 28.7%, 22.3% and 10.5%, respectively.

**Figure 4:**
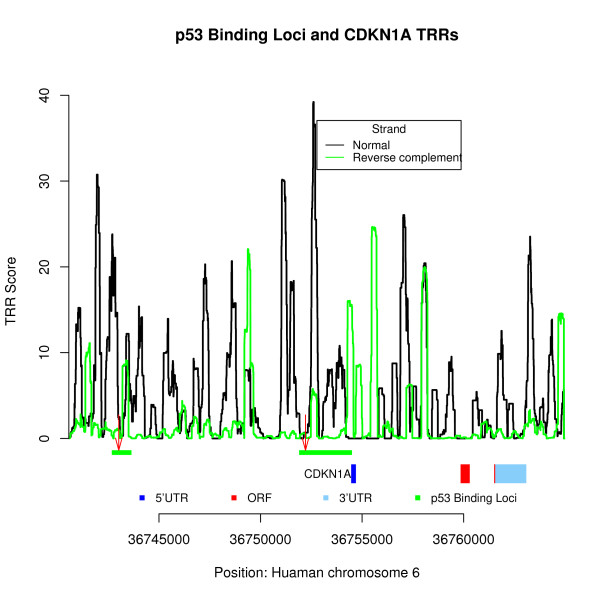
**Core TRRs analysis of the CDKN1A gene in human**. The x-axis represents the genomic positions of the CDKN1A gene, and the y-axis represents the core TRR scores from the HSL model using tuple-pair dictionary from *S. cerevisiae*. Two p53 binding loci by ChIP-PET assay are indicated by the green horizontal bars; the two p53 binding sites by ChIP-PCR assay are indicated by the red arrows [39].

We provide an example using the p53 target gene CDKN1A, which has been well characterized. Two p53 binding loci (indicated by the green horizon bars in Figure 4) were identified by ChIP-PET assay within the 12 k bp upstream region of CDKN1A [39]. The two binding sites located around the 11,447 bp and 2,600 bp upstream regions of the CDKN1A TSS are indicated by the red arrows (Figure 4). In addition, a ChIP-PCR assay confirmed the two binding sites with p53 binding activity, and the locus in the 2,600 bp upstream region of CDKN1A showed stronger binding ability than the one around 11,447 bp [39]. We applied the HSL model to analyze the CDKN1A gene, and the core TRR scores are also shown in Figure 4. Two strong core TRR signals around the p53 binding loci were identified and showed consistent results with the experiments: one around 2200 bp and another around 11,447 bp upstream of the TSS of CDKN1A, the latter having a slightly lower core TRR score.

We also applied the HSL model to two other well-known p53 binding genes (S100A2 and PLK2) with known binding sites [39]. Both examples showed that the p53 binding sites are located adjacent to or within the predicated core TRR regions. Furthermore, the experimentally verified TFBSs of p53 on S100A2 and PLK2 are located more than 2000 bp upstream of the TSSs. This again suggests that the functional core TRR may locate far away from its TSSs. It is also interesting to note that about 80% putative p53 binding sites identified by the p53MH algorithm [40] (with their threshold score of 80) in these binding loci are located adjacent to (less than 500 bp) or within our predicated core TRR regions. The complete results of 542 p53 binding loci can be downloaded from .

### TRR prediction for the rice *OsALYL1 *gene and experimental validations

**Figure 5:**
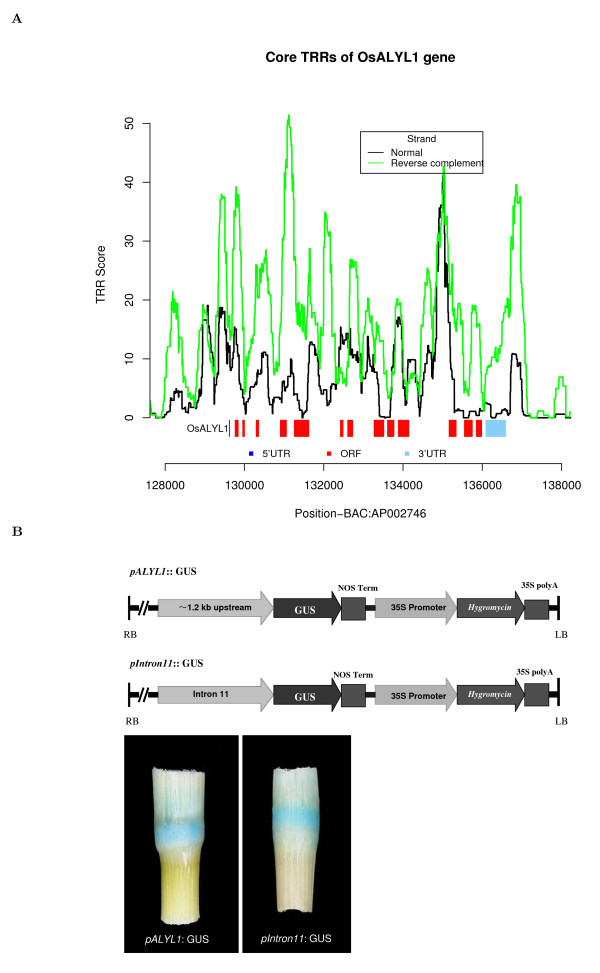
**Core TRRs analysis of the *OsALYL1 *gene and experimental verifications**. (A) The x-axis represents the genomic positions of the *OsALYL1 *(AK064472) gene, and the y-axis represents the core TRR scores from the HSL model using tuple-pair dictionary from *S. cerevisiae*. Significant TRR signals are observed immediate upstream of the TSS of *OsALYL1 *and in intron 11 of *OsALYL1*. (B) The *OsALYL1 *5'-upstream putative promoter region, which contains ~1.2 kb upstream of the coding region, and the full length of its intron 11 were fused to the GUS reporter gene with the nopaline synthase terminator and cloned into the binary vector pCAMBIA1303, respectively. The constructions were named as pALYL1::GUS and pIntron11::GUS, respectively. The GUS staining (blue) was observed at the node of the transgenic lines under the control of both pALYL1::GUS and pIntron11::GUS. This observation suggests that the predicted sequences possess promoter activities in both the upstream of the coding region and intron 11.

ALWAYS EARLY/LIN-9 homologous genes have been shown to play essential roles in the cell cycles of many species, such as *Caenorhabditis elegans *(nematode), *Drosophila melanogaster *and *Homo sapiens *[41-43]. We recently identified an ALWAYS EARLY/LIN-9 homologous gene in *Oryza sativa*, which was named *OsALYL1 *(*Oryza sativa *ALWAYS EARLYLIKE1; in preparation for publication). To analyze the regulation of *OsALYL1*, we first applied the HSL model to the *OsALYL1 *sequence with threshold 20 (in rice, TP = 60.3%, FP = 9.29% for DNA protein coding negative sample and 6.19% for permuted sequences) and found a core TRR signal in the immediate upstream of the TSS of *OsALYL1 *(Figure 5A). Meanwhile, it is interesting to notice that a stronger core TRR signal was also observed in intron 11 of *OsALYL1*.

To experimentally confirm the predictions, the 5'-upstream putative TRR region, which occupies an area ~1.2 kb upstream of the coding region, and the full length of intron 11 were first fused to the GUS reporter gene and were then cloned into binary vector pCAMBIA1303 (Figure 5B). The constructed vectors were introduced into rice respectively to obtain transgenic rice plants (T0 generation). The histochemical assay for GUS activities in the transgenic plants showed that both sequences possess promoter activities (Figure 5B). This finding demonstrates that the predicted sequences possess putative promoter activities in both the upstream of the coding region and intron 11. For details of the experiments, see the **Methods **section. This result also shows that the promoter of a gene can be located in introns. This is consistent with our previous findings, indicating that the promoter of the CKSFL gene in human is in the intron of its upstream gene [44].

### Core TRR and the initiation of microRNA formation

**Figure 6:**
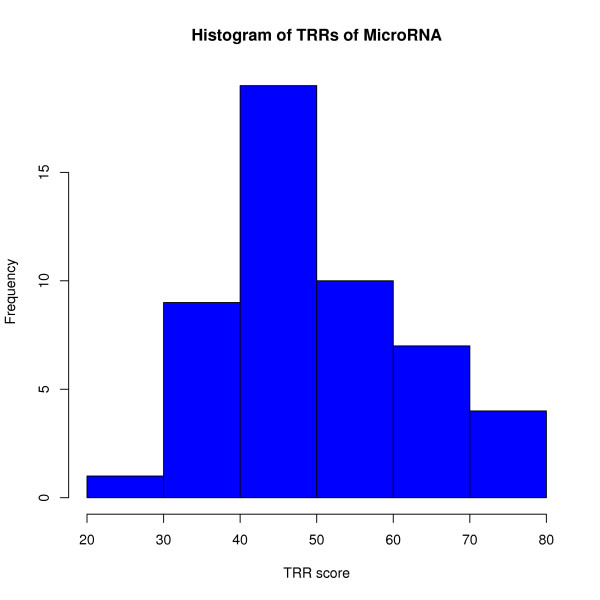
**Core TRRs analysis of microRNAs in *Arabidopsis thaliana***. The HSL model was applied to these 50 microRNAs' 800 bp upstream regions (some are less then 800 bp), and the histogram shows the maximum scores of TRR by HSL in the 800 bp regions of each microRNA.

MicroRNAs are a class of short RNA sequences that play important roles in post-transcriptional gene regulation in complex organisms such as plants and animals. Studies have indicated that microRNA genes possess the same type of promoters as protein-coding genes [19,45,46]. To obtain a more general understanding of the relationship between core TRRs and the regulation of microRNAs, we analyzed upstream sequences from TSSs of 52 microRNAs identified in Arabidopsis via 5'-RACE [19] (the largest dataset of experimentally verified TRRs of microRNAs for a single species to date) by our HSL model. We obtained the 52 microRNA promoter sequences (with lengths ranging from 2 to 800 bp) from [45]. Two promoter sequences less than 400 bp in length were discarded. Forty (80%) out of 50 microRNA upstream regions contain significant HSL scores above 40, and the minimum score was above 20 (Figure 6). After permutation of the 50 promoter sequences, 45 (90%) had scores above 20, while only 13 (26%) of the permutated sequences had scores above 40. For 2000 randomly generated intergenic sequences by RSAT, 94.5% had a score above 20, while only 22.4% had a score above 40. These results indicate that a high threshold is needed for microRNAs of Arabidopsis. Meanwhile, for protein coding DNA sequences of Arabidopsis having the same length as the microRNA promoter sequences, only 15% had a score above 20. This result shows that the upstream regions of the microRNAs are equipped with functional TRRs for the formations of microRNAs. Such result is consistent with previous findings [19,27,45,46].

### Core TRR analysis of *cis*-sense/antisense pairs

Eukaryotic genomes contain many overlapping pairs of oppositely transcribed genes known as *ci*s-sense/antisense pairs [47]. Over 20% of human transcripts are indicated as *cis*-sense/antisense pairs [48]. The proportions of *cis*-sense/antisense pairs in model plants, such as *Oryza sativa L*. and *Arabidopsis thaliana*, are similar to those in human [49,50]. The function and underlying mechanisms of *cis*-sense/antisense pairs are still unclear, except that these pairs tend to be coexpressed [51]. It is therefore natural to hypothesize that there would be a pair of promoters located in the 5' ends of the genes in the *cis*-sense/antisense pairs and that the promoter pairs may control the genes to coexpress [51].

**Figure 7:**
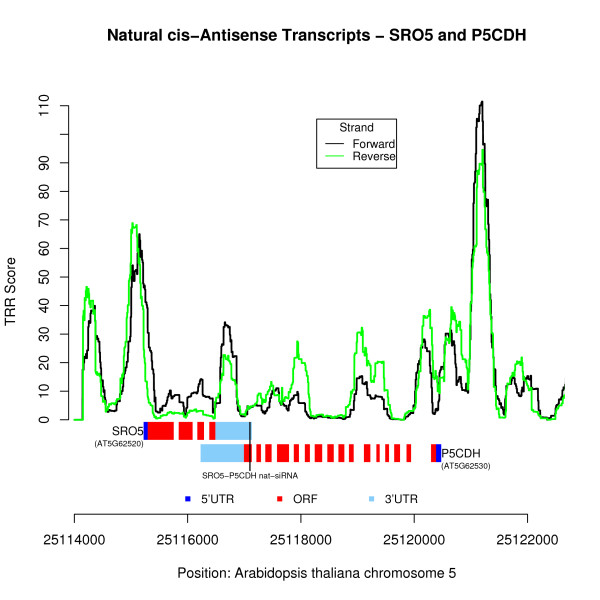
**Core TRRs analysis of *cis*-sense/antisense pair P5CDH and SRO in *Arabidopsis thaliana***. The x-axis represents the genomic positions of the P5CDH and SRO pair, and the y-axis represents the core TRR scores from the HSL model using tuple-pair dictionary from *S. cerevisiae*. The location of 24nt-SRO5-P5CDH siRNA on the DNA sequence is illustrated by the black vertical line.

As an example, we considered the experimentally verified *cis*-sense/antisense pair P5CDH and SRO5 in *Arabidopsis thaliana *[5]. Both of them are essential for the derivation of endogenous siRNAs [5]. The HSL model was applied to scan both P5CDH and SRO5 strands in their regions, and the results are shown in Figure 7. Two significant core TRR signals were predicted by the HSL model, and these are located at immediate upstream of the 5' end of both P5CDH and SRO5. These two signals correspond to the two core promoters of P5CDH and SRO5 genes.

It has been shown that the overlapping transcripts of P5CDH and SRO5 in an antisense orientation form double-stranded RNA (dsRNA) that could further generate both 24-nt and 21-nt short interfering RNAs [5]. The overlapping regions of P5CDH and SRO5 are in the last exons (mainly in 3' UTR) of both genes. Our algorithm predicted a core TRR signal within the overlapping region with maximum TRR scores around 35. The formed siRNA is adjacent to this core TRR region. The 24nt-SRO5-P5CDH siRNA is produced via a biogenesis pathway requiring DCL2, RDR6, SGS3 and NRPD1A with the induction of SRO5 by salt stress. The 24nt-SRO5-P5CDH siRNA may be formed with the help of the nearby core TRR we predicted. Such results can help us to further understand the structure of *cis*-sense/antisense pairs since the overlapped sequences may have essential regulatory functions.

### Examining the regulatory role of human non-coding DNA regions

An increasing number of non-coding DNA regions (including intergenic regions and introns), previously regarded as "junks", have been shown to be apparently functional [52,53]. Although protein coding sequences are only ~1.2% of human genome, it was indicated that at least one-third of the human genome is represented by regulatory sequences for transcript expression [53]. To gain insight into the functions of the non-coding regions, we first studied the TRRs of ncRNAs, most of which are widespread in the non-coding DNA region. We predicated core TRRs on 685 human microRNAs from miRBase [54] (only a small part of them are located in ENCODE regions since they are from the whole human genome) and 6587 putative ncRNAs in ENCODE regions [55]. We took a region 800 bp upstream of each end of the ncRNAs as candidate regulatory regions. (Most ncRNAs are derived from the hairpin precursors; hence, both ends of the ncRNAs may be functional [54]). For each ncRNA, if either candidate regulatory region from one end has a TRR score above a given threshold, then the ncRNA is predicated to have putative TRRs. By applying this rule with a threshold of 20, we found that 63.6% of 685 human microRNAs from miRBase and 63.2% of 6587 putative ncRNAs from ENCODE regions had putative TRRs in their candidate regulatory regions. These results are greater than the corresponding percentage (52.3%) in the (-1500, +100) regions of human EPD genes (see subsection "Core TRRs of the p53 gene" above). We also found that the HSL scores of TRRs for non-coding RNAs tend to be higher than scores for protein coding genes (data not shown), which therefore indicates that TRRs of ncRNAs are more conserved than TRRs of protein coding sequences. This finding is consistent with recent observations that upstream sequences of ncRNAs are more conserved than those of protein-coding genes among mammals [56,57].

We scanned the ENCODE human genome nucleotide regions by HSL and predicted putative TRRs with a threshold 20. There are a total of 44 ENCODE regions, and for all the 44 ENCODE regions [4], the percentages of predicated putative TRR regions vary from 6.2% to 50.8%, with mean 24.7% and standard deviation 12.0%. A total of 22,221 TRRs was predicated in ENCODE regions, and these data are summarized in Additional file 2. Considering that over 10,000–20,000 ncRNAs have been predicated in ENCODE regions (this number is still not completed) [55,58], we postulate that most of our predicted TRRs in the non-coding regions may play essential roles in the regulation of ncRNAs.

## Discussion

Genomic annotations of core promoters (~80–100 bp surrounding the TSSs) are of great importance to the understanding of transcriptional regulation, and numerous methods have been developed to identify core promoters [9,59-61]. High throughput technologies through expressed sequence tag cDNA/ETS/mRNA/CAGE sequences have been developed that can identify TSSs with high accuracy [30,62,63]. Recent computational methods, such as EP3, which utilizes DNA structure features (e.g., GC content and chemo-physical properties of DNA) [28], and CoreBoost_HM, which integrates histone modification information [64], have been implemented and have achieved great improvements. However, few methods have been developed to identify functional regulatory DNA regions genome wide. For example, the EPD database, one of the main promoter databases, does not provide the real functional regulatory regions (TRRs, as we have termed them here), but rather self-setting regions around these experimentally verified TSSs [24,65]. In this study, we found that many functional regulatory regions are located far away from the TSSs or in the intron of genes. This finding is consistent with the observations by others [29,44]. Hence, our HSL model may serve as an additional tool to supplement such computational methods as EP3 [28] and CoreBoost_HM [64] for genomic annotations of potential DNA regulatory regions.

The fact that the number of experimentally verified samples of TRRs is limited makes statistical analysis of TRRs difficult. For this reason, core promoters have been widely studied, and the regions surrounding them may be good candidates for TRRs. However, for genomes of higher eukaryotes, core TRRs are often located in very long intergenic regions or even unknown regions. Moreover, higher eukaryotic genes may frequently be regulated from multiple alternative promoters. For these reasons, we chose the *S. cerevisiae *genome as the model system in our study because it contains relatively short intergenic regions (median length shorter than 400 bp) and a small number of introns [66]. Hence, the intergenic regions in yeast are mostly regulatory regions and can be easily used as a proxy for the sample of TRRs [1].

The concept of core TRR, which we defined earlier, is the foundation of the studies conducted in this paper. Core TRR is similar to *cis*-regulatory module (CRM) in that both characterize functional regions by a cluster of regulatory elements. The main difference is that CRM is characterized by a module consisting of function/gene-specific elements, while the core TRR is a cluster consisting of the common and conserved parts of TRRs. In order to identify CRMs, this difference leads to the need for algorithms to obtain information for sets of co-regulated genes. In contrast, our HSL model requires no such a priori information to elucidate the specific function of genes with reasonably high accuracy. Among the many reasons for this is the fact that TRRs are generally shared by many genes. Therefore, there is no need to pre-select sets of co-regulated genes. In addition, core TRRs are conserved, not only among genes in closely related species, but also across species. We showed that the vocabulary and scoring system of HSL, trained based on sequences of *S. cerevisiae*, can be applied to other organisms, such as *Drosophila*, rice or human, with reasonable accuracy.

Once core TRRs, the conserved parts of TRRs, are obtained, their surrounding regions may be considered candidates of the TRRs. Thus, we can conduct further research to study gene-specific and/or organism-specific regulatory elements around TRRs. For example, p53 binding sites for the genes regulated by the p53 genes are usually located around TRRs and thus cooperate with other elements in the TRR region to exert their functions. Since single TFs cannot function alone, but often function through cooperation with other elements in the TRR, it is worth noting that TRR information can help us eliminate nonsense matches of binding sites in genomic searching.

The HSL model proposed for the identification of TRRs in this study differs from existing approaches in several ways. Unlike most probability models which assume certain models of the underlying sequence, e.g., certain Markov properties, HSL takes the approach of a simple scoring system based on word counting for each layer and excludes most vocabularies that do not appear frequently in the sample sequences. If the number of samples is sufficient, more layers of the HSL model can be added one by one until the model reaches a high enough accuracy for the identification of TRRs. For example, the HSL model using a single tuple dictionary performed poorly everywhere in the region in predicting core TRRs of the p53 gene in human, as shown in Figure 3B. In contrast, the HSL model using tuple-pair dictionary (second layer) identified 10 significant signals with scores higher than 20, as shown in Figure 3A. Among them, six are consistent with current biological understanding. Another advantage of the HSL model is that the computational program is fast and easy to implement.

Although the HSL model provides a useful tool to predict potential core TRRs based only on DNA sequence information, it may still yield high FP rates, especially for complex organisms, such as *Homo sapiens*. The available high throughput epigenetic information showed that functional TRRs are associated with a number of chromatin modification signals [21,36,37]. The information from high throughput assays on chromatin modifications and protein binding was used as evidence to support the predicated potential TRRs in ENCODE regions [67]. Histone modification has also been utilized, which has greatly improved the performance of TSS identification [64]. In this study, this kind of information was used to support TRRs predicted in the p53 gene and p53 target genes. As additional examples, the TRR signals in the first intron of the p53 gene and TRRs in both the upstream and inside regions of the CDKN1A gene were further supported by chromatin modifications [37] and other information (see Additional file 1). The intricate mechanisms between TRRs and chromatin modifications should be the subject of future studies.

Since the non-coding DNA sequences are mostly transcribed and remain to be annotated, it is difficult to obtain real "non-TRR" regions to estimate the false positive rate of HSL in the non-coding regions. It may be even harder to obtain real "non-TRR" regions than to verify TRR regions since many TRRs only function at specific conditions. Even the use of randomly generated intergenic regions by RSAT to obtain "non-TRR" regions may not achieve satisfactory results. This was observed in our study of human that had relatively high false positive rates (Figure 2B). A possible explanation for this result may lie in the heterogeneity of human sequences (mixtures of GC-rich and GC-poor regions), as well as the inability of RSAT to generate accurate human intergenic sequences. Similar problems with evaluations of motif-finding methods were also observed when using random sequences by RSAT (see ). Meanwhile, our results of p53 gene indicate that the false positive rate of our method is not high since most predictions in p53 genes are supported by biological evidence.

Recent studies showed that more than 93% of ENCODE-analyzed human genome nucleotides are transcribed in different cells [4]. In addition, the genomes of all eukaryotes studied are almost entirely transcribed, generating an enormous number of non-protein-coding RNAs (ncRNAs), many of which play a major role in regulation [3,4,68]. These ncRNAs are transcribed separately by their own promoters (TRRs), and they are regulated by regions with core parts similar to those core TRRs for protein-coding transcripts [19,45,46,69-71]. Thus, it is not surprising to find that the core TRR regions can be located pervasively in the genomes [72]. Meanwhile, for human, a number of statistical analyses have already revealed that non-coding sequences (randomly selected sequences) are hierarchically organized and convey more biological information than protein-coding sequences [73,74]. Our results indicated that TRRs are located pervasively in the genomes, and most TRRs might be responsible for the regulation of ncRNAs; the TRRs of ncRNAs are more conserved than those of protein coding genes.

## Conclusion

Evidence shows that genomes of eukaryotes are almost entirely transcribed, generating a large number of protein coding genes and an enormous number of ncRNAs. To reveal the underlying regulatory mechanisms of these transcripts, we proposed the concept of core TRR which refers to a DNA region that contains a cluster of conserved regulatory elements commonly occurring in the majority of regulatory sequences that are essential for the expression of transcripts. We then constructed a hierarchical stochastic language (HSL) model for TRRs in yeast based on regulatory cooperation among core TRR elements. We found that the HSL model trained based on yeast achieved comparable accuracy in predicting TRRs in other species, thus demonstrating the conservation of TRRs across species. Examples such as p53 or *OsALYL1*, as well as the microRNAs, indicate that the identified core TRRs by HSL are highly accurate across species and can serve as proper candidates for the further scrutiny of specific regulatory elements and mechanisms. Our results also showed that core TRRs of protein coding genes and ncRNAs are located pervasively in the genomes and similar in structure, while the latter are more conserved than the former.

## Methods

### DNA sequence preparations

A total of 2,961 sequences extracted from the region (-500, +100) of 2,961 genes (with verified open reading frame (ORF)) located on chromosomes I-XII of *S. cerevisiae *were set as the putative TRR dataset for our algorithm. Ten yeast-coding DNA sequence (CDS) datasets with 10,000 sequences 601 bp in length were randomly sampled from the yeast-coding DNA sequences. Ten random DNA sequence (RDS) datasets with 10,000 sequences 601 bp in length were generated by independent and identical distribution with the same nucleotide frequencies as the yeast genomic sequences. All the *S. cerevisiae *DNA sequences and gene features of ORF were downloaded from the Saccharomyces Genome Database (SGD) (, version: Dec 2005). The 5,015 TSS annotations for yeast genes were from Lee et al. [23].

The putative promoter sequences (annotated TSSs) of 1921 *Drosophila melanogaster*, 1844 *Homo sapiens *genes and 13,044 *Oryza sativa *genes were downloaded from the Eukaryotic Promoter Database (EPD) [24]. The protein coding DNA sequences of *Drosophila melanogaster *were retrieved from the Ensemble database  by BIOMART; the protein coding DNA sequences of *Homo sapiens *were downloaded from the CCDS database at the NCBI (version: Hs35.1, ); and protein coding DNA sequences of *Oryza sativa *were downloaded from the TIGR Rice Genome Annotation database .

The human p53 gene sequence was downloaded from the UCSC Genome Browser with identification number hg17. The annotation of p53 binding loci was from [39], and the corresponding sequences were also retrieved from the UCSC Genome Browser.

The annotation of 685 human microRNAs was from miRBase [54] (version 11.0), and the annotation of 6587 putative ncRNAs in ENCODE regions was from [55]. The corresponding sequences were retrieved from the UCSC Genome Browser with identification number hg18.

The protein coding DNA sequences of *Arabidopsis thaliana *were downloaded from the TAIR database . The promoter sequences of 50 microRNAs of Arabidopsis were downloaded from .

The random intergenic sequences of *Drosophila melanogaster*, *Homo sapiens *and *Arabidopsis thaliana *were generated by Regulatory Sequence Analysis Tools (RSAT) .

### The HSL model Algorithm

The HSL algorithm consists of three steps. First, the k-tuples (k = 6) that are over-represented in the putative TRR dataset, while under-represented in both the CDS datasets and the RDS datasets, are selected as the single-tuple dictionary (SD). For each k-tuple W, we count its occurrences on putative TRR dataset N(W). We approximate their occurrences on the coding DNA sequence (or random DNA sequence) by the normal distribution *N*($\hat{N}$(*W*), $\hat{V}$(*W*)), and the z-score [75] is given by

(1) $$Z(W)=\frac{N(W)-\hat{N}(W)}{\sqrt{\hat{V}(w)}},$$

where $\hat{N}$(*W*) and $\hat{V}$(*W*) can be estimated from the CDS datasets (or RDS datasets). A k-tuple is kept in the SD dictionary if its p-value is less then 0.05.

Second, we consider cooperation between the k-tuples in SD, which are grouped into pairs. The pairs that are over-represented in the putative TRR dataset, while under-represented in the CDS datasets and the RDS datasets, are selected as tuple-pair dictionary (PD) by the same procedures as those for single k-tuple. For each pair P_i _(i stands for the i^th ^pair in the PD), a score is defined as

(2) $$s({\text{P}}_{\text{i}})=\log(\frac{\text{Prob}({\text{P}}_{i}|\text{TRR})}{\text{Prob}({\text{P}}_{i}|\text{CDS})})$$

Third, for a given sequence, we scan the sequence using windows of width L. For each position k on the given sequence, a score is given by

(3) $$T(k)=\sum_{i=1}^{N}s({\text{P}}_{\text{i}})\cdot 1_{\left(p_{i}\in \text{window}[k-\frac{L}{2},k+\frac{L}{2}]\right)}({\text{P}}_{i})$$

as its core TRR score. N is the number of pairs in PD, and 1 is an indicator function. A region on the given sequence having a high score indicates that this region is rich in motif pairs and has a high probability of being a core TRR. A suitable threshold is suggested in the **Results **section.

### p53 binding loci analysis

The p53MH program was downloaded from . For each input sequence, the p53MH algorithm would give the top 3 highest scoring binding sites. We set a loose threshold score of 80 [40] in order to include more potential binding sites. A p53 binding locus with a predicted score greater than 80 was considered to contain a potential binding site in the binding locus.

### GUS activity under the control of the *OsALYL1 *predicted core promoter regions

According to the results of TRR prediction (Figure 5A), the *OsALYL1 *(AK064472) 5'-upstream putative promoter region, which contains ~1.2 kb upstream of the coding region, and the full length of its intron 11 were fused to the GUS reporter gene with the nopaline synthase terminator and then cloned into the binary vector pCAMBIA1303, respectively. In this paper, the constructions were named as pALYL1::GUS and pIntron11::GUS, respectively (Figure 5B). The constructions were introduced into rice variety Taipei309 (*Oryza sativa L*. cv. TP309), respectively, to generate transgenic lines. Procedures for rice tissue culture and transformation with *Agrobacterium tumefaciens *were as described in [76]. Histochemical assay for GUS activity in transgenic plants (T0 generation) was performed as described in [77].

### HSL web interface

A web interface for our HSL model is available from .

## Abbreviations

CDS: coding DNA sequence; CRM: *cis*-regulatory module; HSL: hierarchical stochastic language model; ncRNA: non-protein-coding RNAs; PD: tuple-pair dictionary; RDS: random DNA sequence; SD: single-tuple dictionary; TF: transcriptional factor; TFBS: transcriptional factor binding site; TRR: transcriptional regulation region; TSS: transcriptional starting site.

## Authors' contributions

MQ and MD initiated the study. LW conducted the computational analysis and implemented the algorithm. WF, MD, FS and MQ helped with the computational design and algorithm development. LW, WF, MD, FS and MQ wrote the manuscript. DL contributed to some biological implications of the results. Biological experiments were conducted by DL, DZ, XL and LZ. All the authors read and approved the final manuscript.

## Supplementary Material

Additional file 1 **Supplementary Text.** Comparison results of TRR with gene transcriptional rates in *S. cerevisiae *and core promoter regions for human; biological evidence for identified TRRs of the p53 gene and the CDKN1A gene. Click here for file

Additional file 2 **TRRs in ENCODE regions.** Predicated TRRs by HSL in ENCODE regions. Click here for file
