# Supplementary material for: Conservation and implications of eukaryote transcriptional regulatory regions across multiple species
Source: BMC Genomics. 2008 Dec 20;9:623. doi: 10.1186/1471-2164-9-623 (PMC2640395; doi:10.1186/1471-2164-9-623)
Supplement: Additional file 1 — Supplementary Text. Comparison results of TRR with gene transcriptional rates in S. cerevisiae and core promoter regions for human; biological evidence for identified TRRs of the p53 gene and the CDKN1A gene. [file 1471-2164-9-623-S1.doc]

**Supplementary Text**

**Core TRR scores vs. gene transcriptional rates in *S. cerevisiae***

It is well known that the gene transcriptional rates (GTRs), the number of mRNA produced per unit time, is controlled by their promoters [1]. We used the GTR data in [2] to study the relationship between core TRR scores (the maximum score for both strands in (-500,+100)) and GTRs in yeast. The GTR information is available for 3553 genes with annotation “Verified” and with gene regions of more than 400bp for the whole genome of *S. cerevisiae* [2]. We first filtered out those genes with GTR<1, resulting in 2592 genes, since GTRs less than 1 are not reliable. Figure S1(a) shows the scatter plot of GTRs and the HSL scores together with the loess smoothing curve. It shows that the core TRR score (>20) is positively correlated with GTRs.

We next determine the threshold of core TRR score. For each value T, genes are divided into two groups, the upper score group with core TRR score >T and lower score group with core TRR score ≤ T. The Kolmogorov-Smirnov test was used to detect the difference between the distributions of GTRs in the two groups. Figure S1(b) shows the relationship between the p-value and T. The p-value peaks at T=13 when the two distributions are most likely to be the same and decreases to below 0.01 when T≥20. This suggests that a reasonable threshold for the core TRR score is around 20. This is also illustrated by the boxplots in Figure S1(c), where the 2592 genes are divided into seven classes based on the core TRR scores. It is shown that the distributions of the classes with TRR scores less than 15 and greater than 20 are significantly different.

For the relationship between HSL signals and transcriptional rates, we found that some of those genes with low transcriptional rate have high HSL scores. This may not be a false positive signal of HSL since TFs can act as activators to increase the transcriptional rate of a gene, or as repressors to suppress the transcriptional rate. In the latter case, the transcriptional rate may be low [1]. However, in the case where genes have high transcriptional rates with low HSL scores, it is more likely that HSL misses a target.

**Biological evidence for identified TRRs of the p53 gene and the CDKN1A gene**

The available high throughput epigenetic information shows that functional TRRs are associated with a number of chromatin modifications signals [3-5]. The information from high throughput assays on chromatin modifications and protein binding was used as evidence to support the predicated potential TRRs in ENCODE regions [6]. In the main text, such information was used to support TRRs predicted in the p53 gene and p53 binding loci.

Here, recently generated data of chromatin modifications [3] and other information, such as conservations by multispecies alignments, were used to further support TRR signals in intron 1 of the p53 gene (Figure S2(a)) and TRRs in both upstream and inside regions of the CDKN1A gene (Figure S2(b)). Twenty histone lysine and argininemethylations, as well as histone variant H2A.Z,RNA polymerase II and the insulator binding protein CTCF across the human genome, were generated by high throughput assay [3]. Conservation information by multispecies alignments from the UCSC Genome Browser were also used as sequence evidence since stringently constrained noncoding sequences can be functional [6].

We used two thresholds, 20 (TP=52.3%; FP= 26.7% for DNA protein coding negative sample, 5.21% for permuted sequences and 12% for randomly generated intergenic sequences (Figure 2b)) and 15 (TP=65.9%; FP= 36.4% for DNA protein coding negative sample, 9.66% for permuted sequences and 31.35% for randomly generated intergenic sequences (Figure 2b)).

Both thresholds show that, in intron 1 of the p53 gene and upstream and inside regions of the CDKN1A gene, most of the predicted TRR regions are located in or near some of the chromatin modifications signals and/or conservation signals (Figure S2). This evidence shows that the TRR regions we predicted may relevant biological functions.

**TRR analyses in core promoter regions for human**

Core promoters are usually defined as regions of ~80–100 bp surrounding the TSS. Identification of core promoters is very important for genomic annotation and numerous methods have been developed to identify core promoters with high accuracy, including two recent methods (EP3 [7] and CoreBoost_HM [8]). The methods of TSS identification can greatly benefit from the conservations around core promoters.

Different from core promoter identification, our HSL model aims at identifying functional regulatory regions which may differ from TSSs. For illustration purpose, we restricted the regions of EPD for human to [-100, +100] and found with the criterion based on the ROC curves that the regions [-100, +100] are not good as other regions tested (Figure S3). It does not imply that our method is not valid or the regions [-100, +100] of TSSs are not conserved, but rather that evidence from studies of histone modifications indicates that our method identified functional regulatory regions. For example, in human, the histone modifications of both H3K4me3 and H2A.Z are enriched in active and silenced promoter regions (functional DNA regions) [8]. Figure 1 of [8] shows that H3K4me3 and H2A.Z signals are most depleted in region [-100,+100] and have peaks both upstream and downstream of TSSs. Figure 1 of [8] indicates that the real functional regulatory regions are not restricted to [-100,+100]. This finding is consistent with our results (Figure S3) that when extending the regions from [-100,+100] to [-1500,+100], more functional regions are included and our ROC curves yielded larger AUC and increased power.

**References**

1. Alon U: **An introduction to systems biology : design principles of biological circuits**. Boca Raton, FL: Chapman & Hall/CRC; 2007.

2. Holstege FC, Jennings EG, Wyrick JJ, Lee TI, Hengartner CJ, Green MR, Golub TR, Lander ES, Young RA: **Dissecting the regulatory circuitry of a eukaryotic genome**. *Cell* 1998, **95**(5):717-728.

3. Barski A, Cuddapah S, Cui K, Roh TY, Schones DE, Wang Z, Wei G, Chepelev I, Zhao K: **High-resolution profiling of histone methylations in the human genome**. *Cell* 2007, **129**(4):823-837.

4. Pokholok DK, Harbison CT, Levine S, Cole M, Hannett NM, Lee TI, Bell GW, Walker K, Rolfe PA, Herbolsheimer E *et al*: **Genome-wide map of nucleosome acetylation and methylation in yeast**. *Cell* 2005, **122**(4):517-527.

5. Roh TY, Cuddapah S, Cui K, Zhao K: **The genomic landscape of histone modifications in human T cells**. *Proc Natl Acad Sci U S A* 2006, **103**(43):15782-15787.

6. King DC, Taylor J, Zhang Y, Cheng Y, Lawson HA, Martin J, Chiaromonte F, Miller W, Hardison RC: **Finding cis-regulatory elements using comparative genomics: some lessons from ENCODE data**. *Genome Res* 2007, **17**(6):775-786.

7. Abeel T, Saeys Y, Bonnet E, Rouze P, Van de Peer Y: **Generic eukaryotic core promoter prediction using structural features of DNA**. *Genome Res* 2008, **18**(2):310-323.

8. Wang X, Xuan Z, Zhao X, Li Y, Zhang MQ: **High-resolution human core-promoter prediction with CoreBoost_HM**. *Genome Res* 2008.

**Figure Legend**

Figure S1. TRR scores and gene transcriptional rates (GTR) in yeast. (a) Scatter plot of GTRs and TRR score. Blue curve is the loess smoothing curve using points on the plot. (b) For different threshold T, the p-value of the Kolmogorov-Smirnov test, which was used to detect the difference between the distributions of GTRs in the two groups (above and below T). (c) Boxplots of TRR score and gene activity (GTR).

Figure S2. Chromatin modifications [3] and other information, such as conservations vs. TRR signals. (a) p53 gene (b) CDKN1A gene (The figure is generated by the UCSC Genome Browser with identification number hg18; [http://genome.ucsc.edu](http://genome.ucsc.edu/).)

Figure S3: The same as Figure 2(b) in our manuscript, except that we added an additional test on the region [-100,+100].


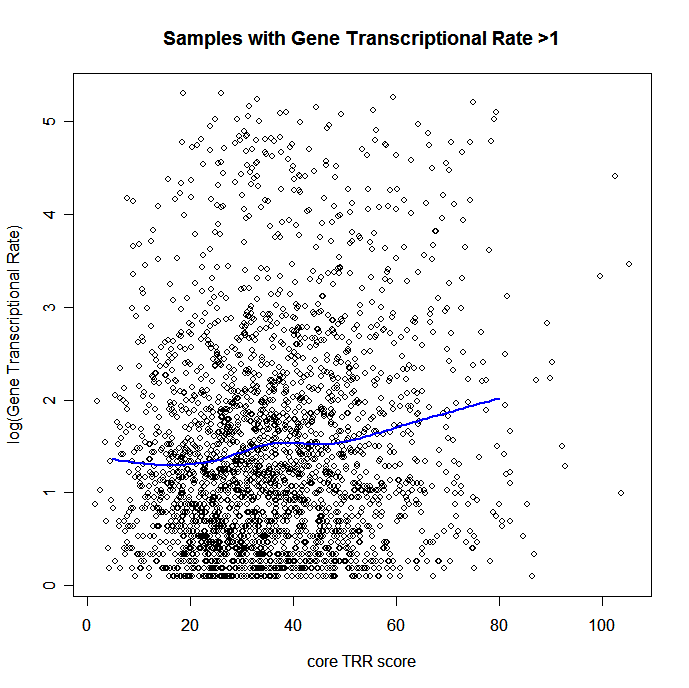


Figure S1(a)


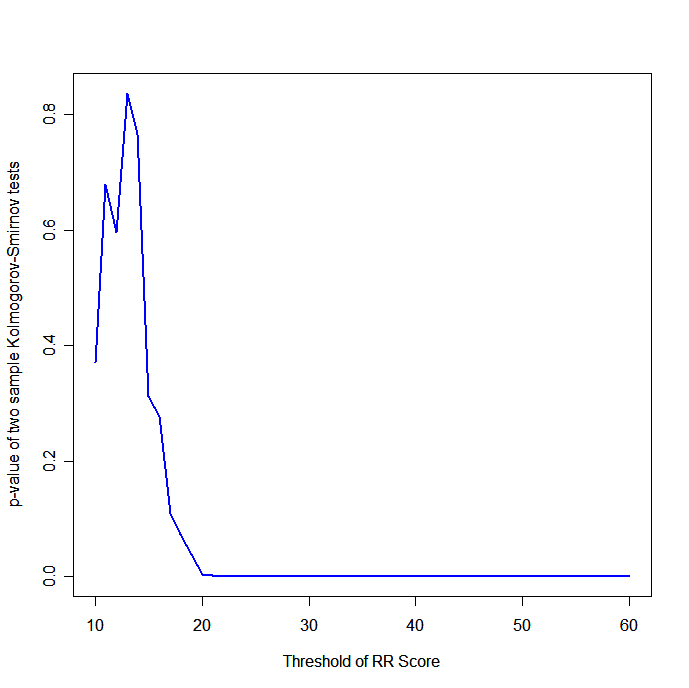


Figure S1 (b)


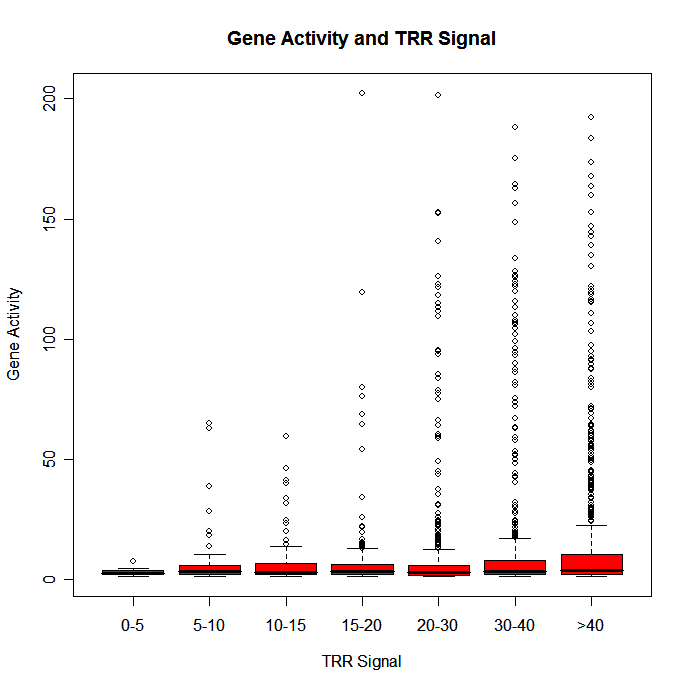


Figure S1 (c)


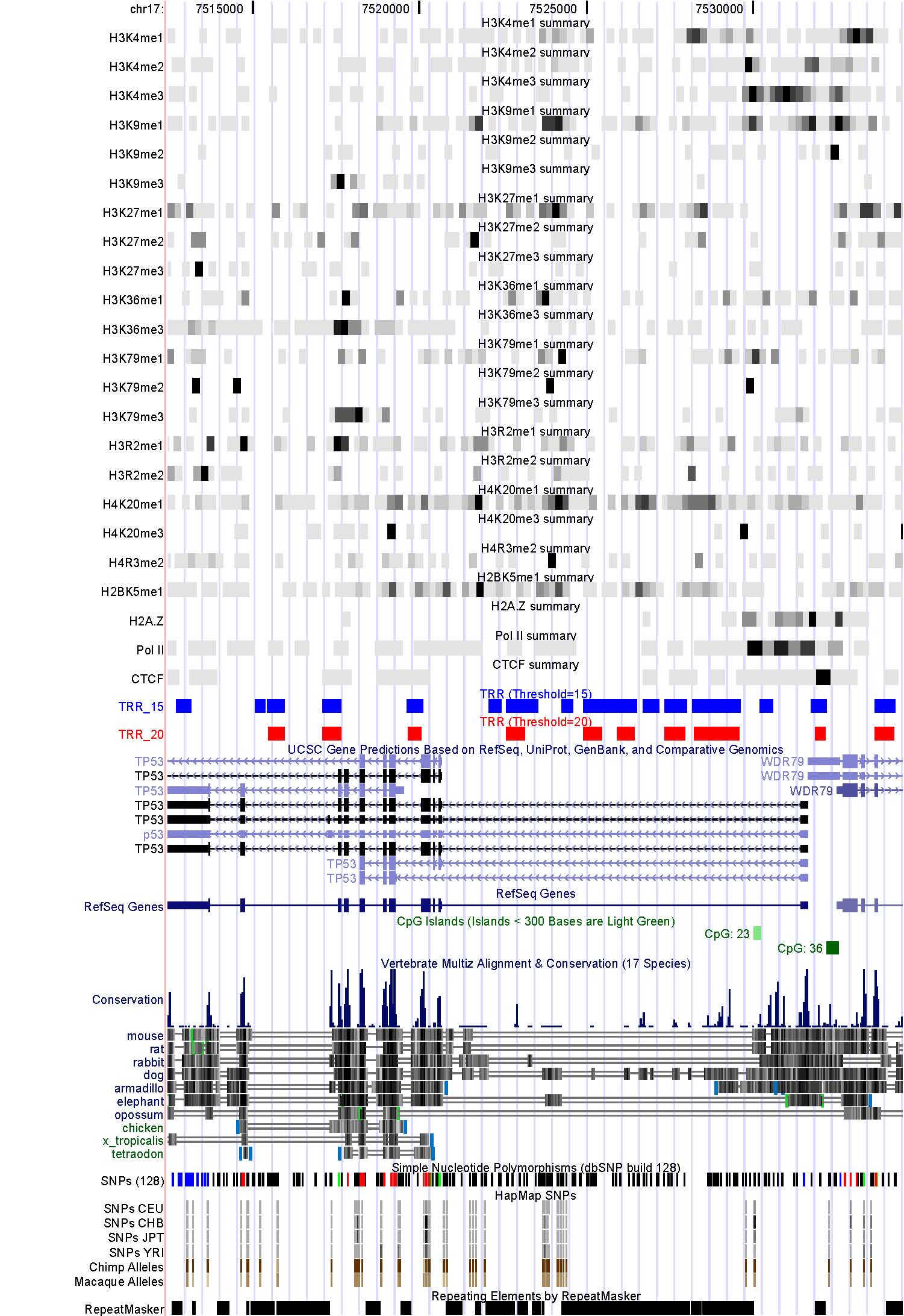


Figure S2 (a)


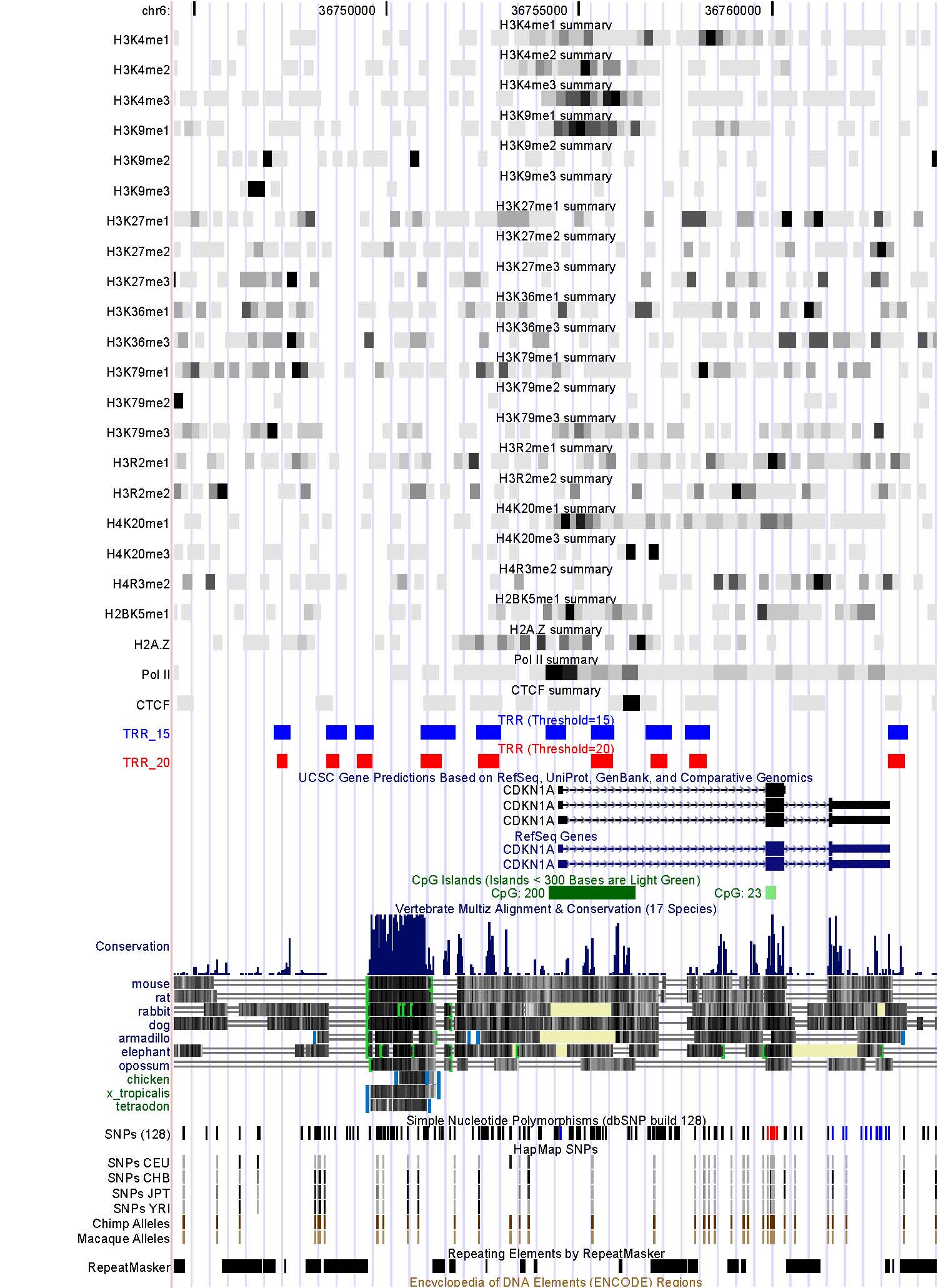


Figure S2 (b)


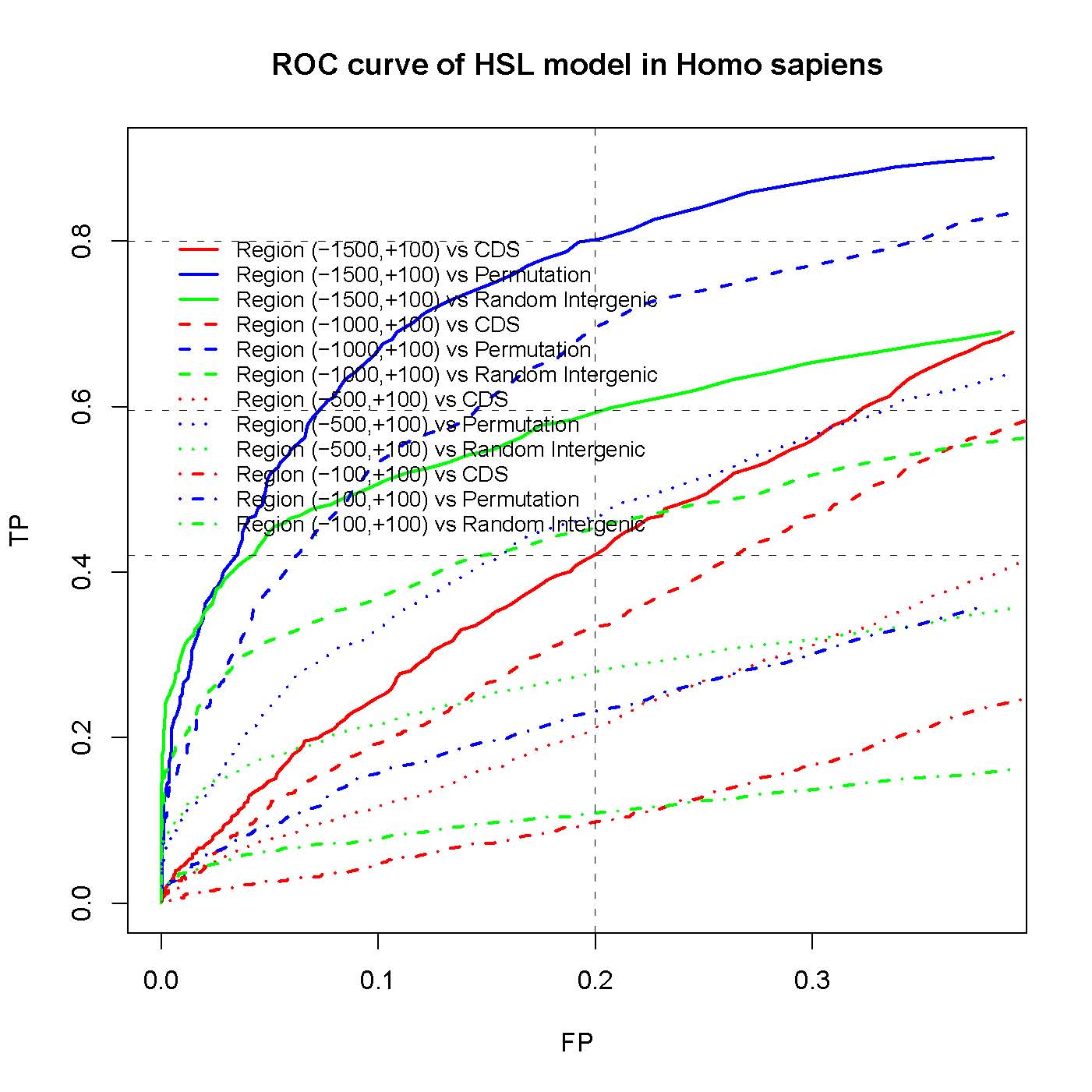


Figure S3
